# Modulation of Properties in [1]Benzothieno[3,2-*b*][1]benzothiophene Derivatives through Sulfur Oxidation

**DOI:** 10.3390/molecules29153575

**Published:** 2024-07-29

**Authors:** Aneta Rzewnicka, Rafał Dolot, Maciej Mikina, Jerzy Krysiak, Remigiusz Żurawiński

**Affiliations:** 1 Division of Organic Chemistry, Centre of Molecular and Macromolecular Studies, Polish Academy of Sciences, Sienkiewicza 112, 90-363 Lodz, Poland; aneta.rzewnicka@cbmm.lodz.pl (A.R.); maciej.mikina@cbmm.lodz.pl (M.M.); jerzy.krysiak@cbmm.lodz.pl (J.K.); 2 Division of Bioorganic Chemistry, Centre of Molecular and Macromolecular Studies, Polish Academy of Sciences, Sienkiewicza 112, 90-363 Lodz, Poland; rafal.dolot@cbmm.lodz.pl

**Keywords:** benzothienobenzothiophene, sulfones, organic electronics, DFT calculations, reorganization energy, fluorescence, semiconductors

## Abstract

This study explores the impact of sulfur oxidation on the structural, optical, and electronic properties of [1]benzothieno[3,2-*b*][1]benzothiophene (**BTBT**) derivatives, specifically focusing on 2,7-dibromo BTBT (**2,7-diBr-BTBT**) and its oxidized forms, 5,5-dioxide (**2,7-diBr-BTBTDO**) and 5,5,10,10–tetraoxide (**2,7-diBr-BTBTTO**). The bromination of **BTBT** followed by sequential oxidation with *m*-chloroperoxybenzoic acid yielded the target compounds in good yields. They were characterized using a wide array of analytical techniques including different spectroscopic methods, X-ray analysis, thermal analysis, and quantum chemical calculations. The results revealed that sulfur oxidation significantly alters the crystal packing, thermal stability, and optoelectronic properties of **BTBT** derivatives. Notably, the oxidized forms exhibited increased thermal stability and enhanced emission properties, with quantum yields exceeding 99%. These findings provide valuable insights for designing advanced organic semiconductors and fluorescent materials with tunable properties, based on the **BTBT** core.

## 1. Introduction

Organic semiconductors (OSCs) have been continuously attracting attention due to their distinctive properties, such as mechanical flexibility and chemical versatility [1]. Their π-conjugated systems can be readily adjusted to optimize molecular arrangement with specific optical and electronic properties [2]. Among these semiconductors, [1]benzothieno[3,2-*b*][1]benzothiophene (**BTBT**) derivatives have been extensively studied and applied in organic electronics due to their favorable characteristics, including high charge carrier mobility, tunable electronic properties, thermal stability, and solution-processability [3,4,5]. Due to these features, **BTBT** derivatives are highly promising materials for different applications, including organic light-emitting diodes (OLEDs) [6,7,8,9,10], organic field-effect transistors (OFET) [11,12,13,14,15,16,17,18,19,20,21,22,23,24,25,26,27], and organic sensors [28,29,30,31,32]. The structural modifications of the **BTBT** core have a profound influence on its electronic properties, intermolecular interactions, and crystal packing, which play a crucial role in the performance of electronic devices [33]. Considering that the presence of thiophene rings in the **BTBT** structure allows for the modulation of its optoelectronic properties through the oxidation of sulfur atoms, this opens up opportunities to develop new materials for organic electronics. Previous studies have demonstrated that the chemical oxidation of the thienyl sulfur atom in thiophene-based poly- and oligomers is a useful strategy to modulate the electronic structure and has an influence on the fluorescence and redox properties [34,35,36]. It was found that oligothiophene *S*,*S*-dioxides are recognized as outstanding candidates for use in light-emitting diodes [37,38,39,40]. Moreover, the oxidation of the sulfur atom in thiophene transforms its electron-donating character into the electron-accepting sulfonyl group. This transformation indicates that oligothiophene sulfones can be interesting from the point of view of their application as bipolar or n-type semiconductors [41,42]. Compared to **BTBT** derivatives, research concerning their oxidized counterparts is very limited, as evidenced by the negligible number of publications in this field. There have been only three publications dedicated to this class of compounds. Two of these focused on the synthesis of **BTBT** 5,5,10,10-tetraoxide (**BTBTTO**) and its reaction with amines [43,44], while one study highlighted the use of diphenylamino derivatives of **BTBT** dioxide and tetraoxide for the fluorescent imaging of lipid droplets [45].

In this paper, we present comparative studies of unoxidized and oxidized **BTBT** derivatives, focusing on the influence of sulfur oxidation on structural, physical, optical, and electronic properties. The investigations were conducted on three compounds: 2,7-dibromo[1]benzothieno[3,2-*b*][1]benzothiophene (**2,7-diBr-BTBT**), its 5,5-dioxide (**2,7-diBr-BTBTDO**), and its 5,5,10,10-tetraoxide (**2,7-diBr-BTBTTO**). The presence of bromine atoms in **2,7-diBr-BTBT** *S*-oxides provides an opportunity for easy further tuning of their desired properties through straightforward functionalization via Heck or Suzuki cross-coupling reactions, paving the way to new compounds with potential optoelectronic applications. To characterize the synthesized compounds, various techniques and methods were employed. Structural verification and analysis of oxidation’s impact on crystal packing were conducted using single-crystal X-ray analysis. Optical properties were investigated through emission and absorption spectroscopy. Thermal stability was assessed using thermogravimetric analysis (TGA) and differential scanning calorimetry (DSC). Additionally, electronic properties, such as frontier orbital energies, ionization potential, electron affinity, and internal reorganization energy, were predicted using DFT and TDDFT methods.

## 2. Results and Discussion

### 2.1. Synthesis of ***2,7-diBr-BTBT*** S-oxides

**Scheme 1:**
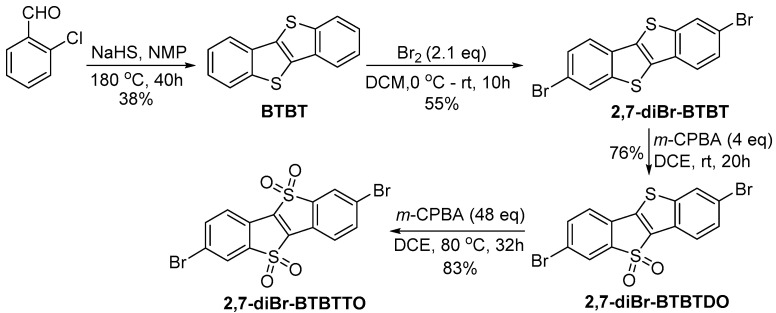
Synthesis of 2,7-diBr-BTBT *S*-oxides.

The synthesis of **2,7-diBr-BTBT** *S*-oxides, as depicted in Scheme 1, commenced with **BTBT** as the starting material, which was obtained using a one-pot procedure from commercially available *o*-chlorobenzaldehyde [46]. Next, **BTBT** was subjected to a bromination reaction to produce **2,7-diBr-BTBT [47]**. The resulting dibromo **BTBT** derivative was oxidized with *m*-chloroperoxybenzoic acid (*m*-CPBA) at room temperature, affording the dioxide **2,7-diBr-BTBTDO** in 76% yield. This compound was subsequently further oxidized to the corresponding tetraoxide **2,7-diBr-BTBTTO**. To avoid issues with separating the product from the unreacted substrate (both compounds have low solubility and similar retardation factors), the reaction was driven to 100% conversion. To ensure this, the reaction was conducted at an elevated temperature with a large excess of *m*-CPBA. The reaction proceeded cleanly, yielding **2,7-diBr-BTBTTO** as the sole product in 83% yield.

### 2.2. Structural Analysis

**Figure 1:**
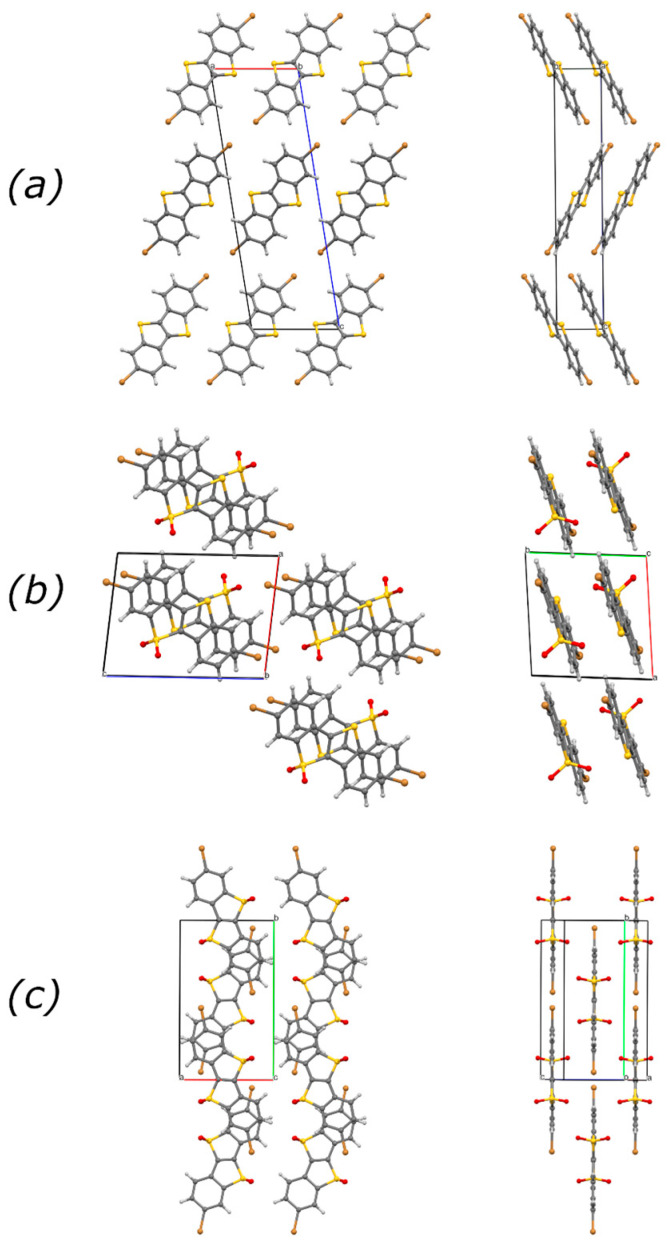
Perspective views of molecular packing in single crystals of (**a**) **2,7-diBr-BTBT**, (**b**) **2,7-diBr-BTBTDO**, and (**c**) **2,7-diBr-BTBTTO**.

X-ray diffraction analysis was performed to confirm the structures of the synthesized **2,7-diBr-BTBT** *S*-oxides. Single crystals of **2,7-diBr-BTBTDO** and **2,7-diBr-BTBTTO** for SC-XRD analysis (CCDC: 2368892 and 2368896) were obtained by heating the dissolved compounds in the corresponding solvents followed by slow cooling of the samples. All crystallographic data are listed in Tables S1–S3.5 in the Supplementary Materials. The analyzed molecules crystallized in different space groups, indicating that the degree of sulfur oxidation has a significant effect on their crystal packing (Figure 1). Furthermore, the crystal packing of the **2,7-diBr-BTBT** *S*-oxides differed from the well-known herringbone arrangement observed for the unoxidized parent compound [47]. **2,7-diBr-BTBT** forms a crystal structure where molecules are π-stacked, maintaining an interplanar spacing typical for flat halogenated OSCs of approximately ~3.46 Å [48]. **2,7-diBr-BTBTDO** and **2,7-diBr-BTBTTO** also crystallize in an π-stacked molecular arrangement, but the oxidation of one or two sulfur atoms has a significant effect on the arrangement of the crystals obtained. **2,7-diBr-BTBTDO** forms π-stacked molecular columns with an alternating arrangement of the oxidized sulfur atoms and an interplanar distance of ~3.55 Å between adjacent molecules. In contrast, **2,7-diBr-BTBTTO** forms oppositely oriented planes with a distance of ~3.46 Å, and each molecule is π-stacked with four neighboring molecules.

The oxidation of the sulfur atoms in **2,7-diBr-BTBT** not only changes the molecular packing but also influences the number of close contacts between the atoms of neighboring molecules. For **2,7-diBr-BTBT**, ten contacts were observed, including two S⋯S and four Br⋯Br contacts per molecule (Table S4.1 in the Supplementary Materials). Oxidation prevents the formation of contacts by sulfur atoms, while oxygen atoms form close contacts, including O⋯H (in both *S*-oxides), O⋯Br, O⋯C(sp2), and O⋯S (only in **2,7-diBr-BTBTDO**). Br⋯Br contacts are also present in **2,7-diBr-BTBTDO**, whereas they are absent in **2,7-diBr-BTBTTO**. The number of contacts varies depending on the oxidation state of the molecules (Tables S4.2 and S4.3 in the Supplementary Materials). Fifteen contacts were listed for **2,7-diBr-BTBTDO**, while their number increased to twenty–four per molecule for **2,7-diBr-BTBTTO**.

### 2.3. Physical and Optical Properties

The obtained compounds were white (**2,7-diBr-BTBT**), yellowish (**2,7-diBr-BTBTDO**), and yellow (**2,7-diBr-BTBTTO**) solids. To evaluate the thermal stability of **2,7-diBr-BTBT** and its oxidized derivatives, thermogravimetric analysis (TGA) and differential scanning calorimetry (DSC) were carried out. The results show that the thermal stability increases with the number of oxygen atoms in the molecule (Table 1 and Figure S1 in the Supplementary Materials). The fully oxidized **2,7-diBr-BTBTTO** undergoes decomposition approximately 18 °C and 38 °C above the decomposition temperatures of its partially oxidized counterpart **2,7-diBr-BTBTDO** and its unoxidized parent compound **2,7-diBr-BTBT**, respectively. In addition, the compounds do not exhibit liquid crystal properties. DSC measurements showed that they do not undergo any phase transitions up to their melting point or decomposition temperature (Figure S2 in the Supplementary Materials).

**Figure 2:**
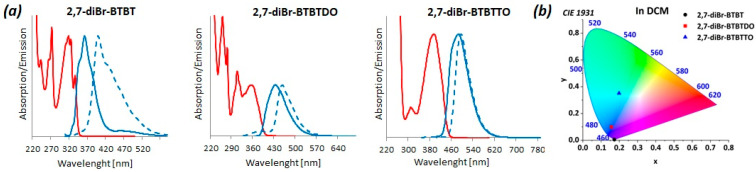
Optical properties of **2,7-diBr-BTBT** and its *S*-oxides; (**a**) Normalized UV–Vis spectra (red solid line—in DCM) and PL spectra (blue solid line—in DCM, blue dashed line—in solid); (**b**) Chromaticity chart.

We performed UV–Vis and photoluminescence (PL) measurements on the synthesized compounds in both solution (dichloromethane (DCM)) and the solid state. The findings, detailed in Table 1 and Figure 2, demonstrate that the oxidation of sulfur atoms within the thiophene rings has a substantial impact on their optical properties. The increase in the number of oxygen atoms in the molecule results in noticeable shifts in both the absorption (λ_abs_) and emission maxima (λ_em_) to the longer wavelengths. Furthermore, the Stokes shift for oxidized derivatives in solution is significantly higher, ranging from 79 to 90 nm, whereas **2,7-diBr-BTBT** exhibits a much smaller shift of about 25 nm. The quantum yield (Ф) of **2,7-diBr-BTBT** is less than 1%, which is lower than the 3.4% quantum yield observed for **BTBT** [49]. This result is in line with the expected heavy atom effect [50,51]. In contrast, for **2,7-diBr-BTBTDO** and **2,7-diBr-BTBTTO**, the quantum yields are significantly higher, surpassing 99% for both compounds. The emission maxima of the investigated compounds in the solid state are in the range of 399–495 nm and exhibit a smaller red shift of 9 to 37 nm in comparison to their emission maxima in solution.

### 2.4. Computational Analysis

**Figure 3:**
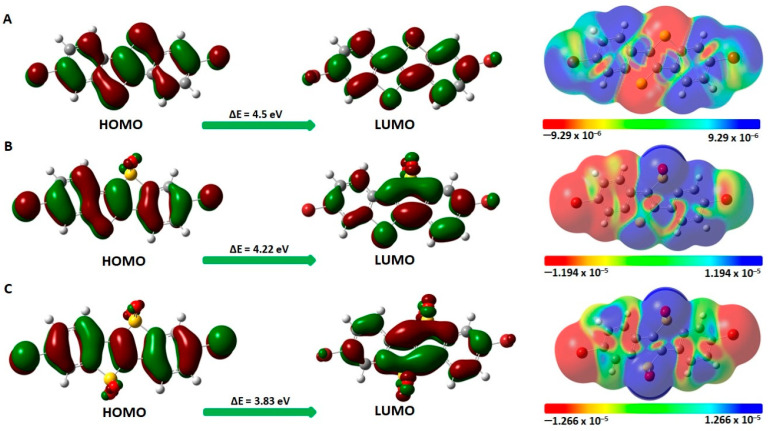
Graphical representation of frontier MOs (isovalue = 0.026) and changes in electron densities during excitation from the ground state to the S_1_ excited state ((**A**)—**2,7-diBr-BTBT**; (**B**)—**2,7-diBr-BTBTDO**; (**C**)—**2,7-diBr-BTBTTO**).

To provide insight into the electronic changes accompanying the oxidation of sulfur atoms in **2,7-diBr-BTBT**, TDDFT calculations were performed using the PBE0/6–311+G(2d,p)//D3-M06-2X/def2-TZVP method. The results of these calculations are summarized in Table 2 and graphically presented in Figure 3. To account for solvent–solute interactions in the calculation of the electronic properties, the IEFPCM model [52,53,54] for DCM was applied. The calculated absorption maxima perfectly match the experimental data, with deviations ranging from 0.06 to 0.14 eV. The largest deviation was observed for **2,7-diBr-BTBTTO**, where the absorption band was red-shifted by approximately 18 nm compared to the experimental value. In all the compounds examined, the lowest energy bands were predominantly associated with the HOMO⟶LUMO transitions (>97%). During the oxidation process, the HOMO energy gradually decreased from –6.230 eV for **2,7-diBr-BTBT** to –7.048 eV for **2,7-diBr-BTBTTO**. The same trend, but to a greater extent, was observed for the LUMO energy. In this case, the energy difference between the LUMO of **2,7-diBr-BTBT** and **2,7-diBr-BTBTTO** reached 1.48 eV. As the energy of the frontier orbitals decreased, the energy band gap also reduced, ultimately reaching around 3.83 eV for **2,7-diBr-BTBTTO**. These results are in strong agreement with the findings reported for thiophene dioxides [36,55] and support our observation that the emission maxima of the investigated compounds exhibit an increasing red shift with a greater number of oxygen atoms. Hole–electron analysis [56] for individual atoms in **2,7-diBr-BTBT** revealed that during excitation to the first excited state, two sulfur atoms and two carbon atoms connecting them provide a considerable input to the hole. The sum of their contribution is almost 50%. During electron excitation, these atoms lose 0.25 electrons, which are transferred to the remaining carbon atoms. The oxidation of thiophene sulfur alters the electron-donating thienyl sulfur atom into a strong electron-accepting group, which is reflected in the electron distribution during excitation. Consequently, in **2,7-diBr-BTBTTO**, the two sulfonyl groups and their connecting carbon atoms gain 0.31 electrons, primarily from the bromine atoms. In the case of asymmetrical **2,7-diBr-BTBTDO**, electron excitation increases electron density at the sulfonyl group and the phenyl ring fused with the thiophene oxidized moiety.

To estimate the ambient stability of investigated compounds and their charge transport properties, the adiabatic ionization potential (IP), electron affinity (EA), and internal reorganization energy (λ) were calculated (Table 3). IP and EA are crucial for understanding the ease with which molecules can gain or lose electrons, directly influencing their electronic properties and reactivity. Furthermore, these parameters also allow us to estimate the energy barriers involved in the injection of holes and electrons into molecules, which is particularly valuable in the design and development of materials for electronic and optoelectronic applications. On the other hand, the hole (λ_h_) and electron (λ_e_) reorganization energies, which are associated with the process of transferring an electron or hole between two adjacent molecules, are the key parameters in the study of charge transport in organic semiconductors. Calculations at the D3-M06-2X/def2-TZVPD level revealed that the IP of the investigated compounds increased with oxidation, from 7.69 eV for unoxidized **2,7-diBr-BTBT** to 8.73 eV for **2,7-diBr-BTBTTO**. These higher IPs for the oxidized **2,7-diBr-BTBT** counterparts indicate greater oxidation stability, making these compounds less susceptible to oxidation-related degradation. The same trend was observed for the electron affinity energy, which gradually increased from 0.72 eV for **2,7-diBr-BTBT** to 2.42 eV for **2,7-diBr-BTBTTO**.

Thiophene sulfur oxidation also alters the internal reorganization energy (the sum of λ_h_ and λ_e_), which increases in the order: **2,7-diBr-BTBT** < **2,7-diBr-BTBTDO** < **2,7-diBr-BTBTTO**. While the values of λ_h_ and λ_e_ are similar for **2,7-diBr-BTBT**, they differ significantly in the oxidized derivatives. For **2,7-diBr-BTBTDO**, the difference between λ_h_ and λ_e_ is about 0.07 eV, suggesting a greater propensity to conduct electrons rather than holes. The opposite situation is observed for **2,7-diBr-BTBTTO**, for which this difference is approximately –0.26 eV, indicating a preference for hole transport.

## 3. Materials and Methods

### 3.1. Synthesis of Materials

#### 3.1.1. General Remarks

Commercial-grade reagents and solvents were used as received without further purification. The ^1^H, ^13^C, and 2D NMR spectra were recorded using Bruker AV Neo 400 or Bruker Avance III 500 spectrometers (Bruker, Billerica, MA, USA). Chemical shifts for ^1^H and ^13^C NMR spectra are reported in parts per million (ppm) relative to the residual proton signal of the deuterated solvent. High-resolution mass spectrometry (HRMS) analyses were conducted on a Waters Synapt HDMS mass spectrometer (Waters Corporation, Milford, MA, USA). The synthesis of **BTBT [46]** and **2,7-diBr-BTBT [47]** was carried out according to established literature procedures. The NMR spectra of all new compounds are included in the Supplementary Materials.

#### 3.1.2. Synthetic Procedures

**2,7-Dibromo[1]benzothieno[3,2-*b*][1]benzothiophene 5,5-dioxide (2,7-diBr-BTBTDO):** A mixture of **2,7-diBr-BTBT** (100 mg, 0.25 mmol) and *m*-CPBA (248 mg, 1 mmol, 4 eq) in DCE (200 mL) was stirred at room temperature for 20 h. The reaction mixture was then washed successively with saturated aqueous solutions of Na_2_SO_3_ and NaHCO_3_, followed by water. The organic layer was dried over anhydrous MgSO_4_. Solvent evaporation, followed by the purification of the crude material by column chromatography using a dichloromethane/petroleum ether (2:1) mixture, afforded **2,7-diBr-BTBTDO** (81 mg, 76%) as a pale yellow solid. Crystals suitable for X-ray analysis were obtained by crystallization from DCE. R_f_ = 0.42 (DCM/petroleum ether, 2:1); ^1^H NMR (500 MHz, CDCl_3_) δ 8.07 (d, *J* = 1.7 Hz, 1H), 7.94–7.88 (m, 2H), 7.76 (dd, *J* = 8.1, 1.8 Hz, 1H), 7.68 (dd, *J* = 8.6, 1.7 Hz, 1H), 7.39 (d, *J* = 8.1 Hz, 1H); ^13^C NMR (126 MHz, CDCl_3_) δ 144.37, 144.16, 143.04, 136.82, 134.11, 130.80, 129.31, 127.09, 126.64, 125.75, 124.83, 123.51, 121.03. HRMS (APCI−) *m*/*z*: calcd. for C_14_H_6_O_2_S_2_Br_2_ 427.8176, found 427.8181.

**2,7-Dibromo[1]benzothieno[3,2-*b*][1]benzothiophene 5,5,10,10-tetraoxide (2,7-diBr-BTBTTO):** To a solution of **2,7-diBr-BTBTDO** (54.8 mg, 0.128 mmol) in DCE (24 mL) stirred at 80 °C, three portions of *m*-CPBA (379 mg, 1.53 mmol, 12 eq) were added at 8 h intervals. After 32 h, the reaction mixture was cooled to room temperature. The precipitate formed during the reaction was filtered off, washed with DCM and diethyl ether, and dried, yielding **2,7-diBr-BTBTTO** (49 mg, 83%) as a yellow solid. Crystals suitable for X-ray analysis were obtained by crystallization from hot DMSO. R_f_ = 0.40 (DCM/petroleum ether, 2:1) ^1^H NMR (500 MHz, CDCl3) δ 7.96 (d, *J* = 1.7 Hz, 1H), 7.85 (dd, *J* = 8.1, 1.8 Hz, 1H), 7.57 (d, *J* = 8.1 Hz, 1H). Due to the very low solubility of **2,7-diBr-BTBTTO** in commonly used deuterated solvents, recording the ^13^C NMR spectra was not feasible. HRMS (APCI−) *m*/*z*: calcd. for C_14_H_6_O_4_S_2_Br_2_ 459.8074, found 459.8079.

### 3.2. X-ray Analysis

Suitable crystals of **2,7-diBr-BTBTDO**, and **2,7-diBr-BTBTTO** were selected, transferred to mineral oil, and mounted on cryo loops. The crystals were then flash-cooled directly in a stream of N_2_. Diffraction intensities were recorded using a Rigaku XtaLAB Synergy-S diffractometer (Rigaku Europe SE, Neu-Isenburg, Germany) equipped with a Cu Kα radiation source (λ = 1.5418 Å) and a HyPix-6000HE hybrid photon counting detector (Rigaku Europe SE, Neu-Isenburg, Germany). The total number of runs and images was based on the strategy calculation of the CrysAlisPro program (Rigaku Oxford Diffraction, v 1.171.43.111a, 2024). The molecular models of the structures were created with the structure solution program SHELXT 2018/2 [57] using intrinsic phasing with Olex2 v.1.5 [58] as the graphical interface and refined by least squares with the 2018/3 version of SHELXL [59]. All non-hydrogen atoms were refined anisotropically. The positions of the hydrogen atoms were calculated geometrically and refined using the riding model. The structures were validated with CheckCif (http://checkcif.iucr.org, accessed on 9 July 2024) and deposited in the Cambridge Crystallographic Data Centre (CCDC) under the accession numbers 2368892 and 2368896 for **2,7-diBr-BTBTDO** and **2,7-diBr-BTBTTO**, respectively.

### 3.3. Optical Characterization

Electronic absorption spectra were recorded on a Shimadzu UV–VIS spectrophotometer UV 2700 (Shimadzu Scientific, Kyoto, Japan). Photoluminescence and quantum yield measurements were performed using a Horiba Scientific FluoroMax+ spectrofluorometer (Horriba Scientific, Kyoto, Japan). All measurements were conducted at room temperature in a 1 cm cuvette. Spectrophotometric grade dichloromethane (DCM) was used for measurements in solution. Photoluminescence spectra of **2,7-diBr-BTBTDO** and **2,7-diBr-BTBTTO** were recorded after excitation at their respective maximum absorption wavelengths, and a 300 nm excitation wavelength was used for **2,7-diBr-BTBT**. Photoluminescence quantum yields (Ф) were determined using an integrating sphere.

### 3.4. Thermal Properties

Thermogravimetric analysis (TGA) was conducted using a TGA 2950 analyzer (TA Instruments, Eden Prairie, MN, USA). The experiments were performed under a nitrogen atmosphere in the temperature range of 50 °C to 600 °C at a heating rate of 10 °C min^−1^. The decomposition temperature was determined at 5% weight loss. The thermal properties of the synthesized compounds were studied using differential scanning calorimetry (DSC) on a DSC 2920 (TA Instruments), heating from 0 °C to 220 °C at a rate of 5 °C min^−1^.

### 3.5. Computational Details

All quantum-mechanical calculations were performed using Gaussian 09, revision D.01 [60]. For simulating optical properties, the geometries of the investigated compounds were optimized using the M06-2X [61] functional with the D3 version [62] of dispersion correction and the Karlsruhe valence triple-zeta def2-TZVP basis set [63]. Calculations were conducted in a dichloromethane (DCM) dielectric medium, applying tight convergence criteria for geometry and energy. The “ultrafine” integration grid was used for all calculations. The character of the equilibrium point on the potential energy surface was identified through frequency calculations. TDDFT calculations were performed for the first six singlet excited states using the PBE0 functional [64] in conjunction with the Polple 6–311+G(2d,p) basis set [65]. Calculations of the internal reorganization energy, adiabatic ionization potential (IP), and adiabatic electron affinity (EA) were conducted at the D3-M06-2X/def2-TZVPD level in the gas phase. Adiabatic IPs were calculated as the energy difference between the relaxed cationic state (E^+^(M^+^)) and the geometry-optimized neutral molecule (E^0^(M)) (IP = E^+^(M^+^) − E^0^(M)). EAs were determined by the energy difference between the optimized neutral molecule (E^0^(M)) and the anionic state (E^−^(M^−^))(EA = E^0^(M) − E^−^(M^−^)). The hole (λ_h_) and electron (λ_h_) reorganization energies were calculated using the following equations:

λ_h_ = λ_1_ + λ_2_ = [E^+^(M) − E^+^(M^+^)] + [E^0^(M^+^) − E^0^(M)] (1)

**Figure 4:**
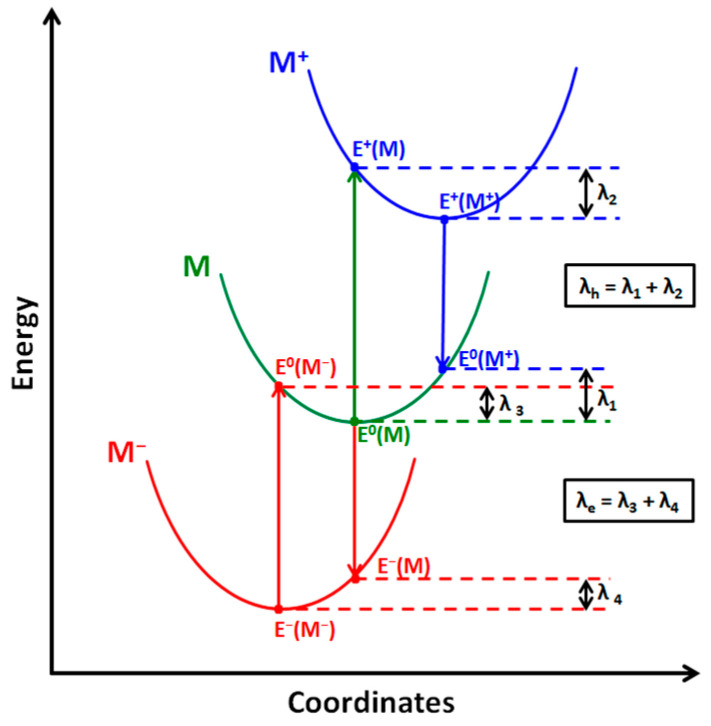
Diagram illustrating the calculation of the hole (λ_h_) and electron (λ_e_) reorganization energies.

λ_e_ = λ_3_ + λ_4_ = [E^0^(M^−^) − E^0^(M)] + [E^−^(M) − E^−^(M^−^)] (2)

where E^+^(M)/E^−^(M) is the energy of the cation/anion calculated at the optimized geometry of the neutral molecule, and E^0^(M^+^)/E^0^(M^−^) is the energy of the neutral molecule calculated at the optimized geometry of the corresponding cation/anion. A graphical plot for the calculation of reorganization energy is presented in Figure 4.

Hole–electron analysis using Hirshfeld partitions was conducted with the Multiwfn 3.8 program [66]. Basis sets def2-TZVP [63] and def2-TZVPD [67] were downloaded from the Basis Set Exchange (BSE) [68]. The optimized geometrical parameters of the investigated compounds are provided in the Supplementary Materials.

## 4. Conclusions

In summary, the sulfone derivative **2,7-diBr-BTBTDO** and the disulfone derivative **2,7-diBr-BTBTTO** have been successfully synthesized via the oxidation of **2,7-diBr-BTBT** with *m*-CPBA and fully characterized using various analytical and theoretical methods. Comparative analysis of **2,7-diBr-BTBT** and its oxidized derivatives revealed the potential of sulfur oxidation as a strategy to tune the properties of **BTBT** derivatives. The oxidized compounds are characterized by red-shifted absorption and emission spectra with a Stokes shift reaching 90 nm and an impressive quantum yield above 99%. The oxidized compounds also show a decrease in both HOMO and LUMO energies, leading to a reduced energy band gap. This increased stability is further supported by their improved thermal properties, which show an increase in decomposition temperature with an increasing number of oxygen atoms. Changes in the electronic properties of the individual molecules also affect their tendency to form unique crystal patterns. Although all investigated compounds form π-stacked molecular arrangements, they differ significantly, as reflected by their different space group symmetry. All the above findings are instrumental in designing new materials based on the **BTBT** skeleton with optimized properties for organic electronic applications and fluorescence-based techniques.

## Figures and Tables

**Table 1:** Physical and optical properties of **2,7-diBr-BTBT** and its *S*-oxides in DCM and solid state.

Compound	DCM				Solid	
	λ_abs_ [nm]	λ_em_ [nm]	Stokes Shift [nm] (cm^−1^)	Ф	λ_em_ [nm]	T_d_ [°C] ^1^
**2,7-diBr-BTBT**	337	362	25 (2049)	<1	399	256.4
**2,7-diBr-BTBTDO**	356	435	79 (5101)	>99	459	296.2
**2,7-diBr-BTBTTO**	396	486	90 (4676)	>99	495	314.5

^1^ Decomposition temperature determined by TGA corresponding to 5% weight loss at 10 °C min^−1^ under nitrogen flow.

**Table 2:** Electronic properties of **2,7-diBr-BTBT** and its *S*-oxides calculated using the TDDFT PBE0/6–311+G(2d,p)//D3-M06-2X/def2-TZVP method in DCM.

Compound	E_HOMO_ [eV]	E_LUMO_ [eV]	Energy Gap [eV]	Excited State	E_ex_^1^ [eV]	λ_ex_ ^2^ [nm]	Oscillator Strength	Major Transitions [%]
**2,7-diBr-BTBT**	–6.230	–1.734	4.496	S_1_	3.762	329.6	0.765	H⟶L (97.4)
**2,7-diBr-BTBTDO**	–6.672	–2.447	4.225	S_1_	3.422	362.3	0.605	H⟶L (97.8)
**2,7-diBr-BTBTTO**	–7.048	–3.218	3.830	S_1_	2.990	414.6	0.560	H⟶L (98.7)

^1^ E_ex_—excitation energy; ^2^ λ_ex_—excitation wavelength.

**Table 3:** Ionization potentials, electron affinities, and reorganization energies of **2,7-diBr-BTBT** and its *S*-oxides.

Compound	IP [eV]	EA [eV]	λ_h_ [eV]	λ_e_ [eV]	λ [eV]
**2,7-diBr-BTBT**	7.69	0.70	0.302	0.328	0.630
**2,7-diBr-BTBTDO**	8.17	1.41	0.390	0.319	0.719
**2,7-diBr-BTBTTO**	8.73	2.42	0.328	0.591	0.919

## Data Availability

The data presented in this study are available in Supplementary Materials.

## Supplementary Materials

The following supporting information can be downloaded at https://www.mdpi.com/article/10.3390/molecules29153575/s1. NMR spectra of all new compounds (Section S1. NMR Spectra); crystallographic data for all investigated compounds (Section S2. X-ray analysis including Table S1. Crystal structure, data collection and refinement parameters of the **2,7-diBr-BTBT** *S*-oxides studied in this research; Table S2.1. Fractional atomic coordinates (×10^4^) and equivalent isotropic displacement parameters (Å^2^×10^3^) for **2,7-diBr-BTBTDO**; Table S2.2. Anisotropic displacement parameters (Å^2^×10^3^) for **2,7-diBr-BTBTDO**; Table S2.3. Bond lengths for **2,7-diBr-BTBTDO** (Å); Table S2.4. Bond angles for **2,7-diBr-BTBTDO** (°); Table S2.5. Torsion angles for **2,7-diBr-BTBTDO** (°); Table S3.1. Fractional atomic coordinates (×10^4^) and equivalent isotropic displacement parameters (Å^2^×10^3^) for **2,7-diBr-BTBTTO**; Table S3.2. Anisotropic displacement parameters (Å^2^×10^3^) for **2,7-diBr-BTBTTO**; Table S3.3. Bond lengths for **2,7-diBr-BTBTTO** (Å); Table S3.4. Bond angles for **2,7-diBr-BTBTTO** (°); Table S3.5. Torsion angles for **2,7-diBr-BTBTTO** (°); Table S4.1. List of intermolecular short contacts in the crystal structure of **2,7-diBr-BTBT**; Table S4.2. List of intermolecular short contacts in the crystal structure of **2,7-diBr-BTBTDO**; Table S4.3. List of intermolecular short contacts in the crystal structure of **2,7-diBr-BTBTTO**); thermogravimetric and differential scanning calorimetry charts (Section S3. TGA and DSC analysis including Figure S1. TGA analysis for **2,7-diBr-BTBT** *S*-oxides; Figure S2. DSC analysis for **2,7-diBr-BTBT** *S*-oxides); geometrical parameters of **2,7-diBr-BTBT**, **2,7-diBr-BTBTDO**, and **2,7-diBr-BTBTTO** optimized at the TDDFT PBE0/6–311+G(2d,p)//D3-M06-2X/def2-TZVP level in DCM (IEFPCM solvation model); atomic coordinates of **2,7-diBr-BTBT** and its *S*-oxides (charged and neutral) optimized at the D3-M06-2X/def2-TZVPD level (Section S4. Theorethical calculations).
