# Supplementary material for: Modulation of Properties in [1]Benzothieno[3,2-b][1]benzothiophene Derivatives through Sulfur Oxidation
Source: Molecules. 2024 Jul 29;29(15):3575. doi: 10.3390/molecules29153575 (PMC11314126; doi:10.3390/molecules29153575)
Supplement: Supplementary file 1 [file molecules-29-03575-s001.zip › 2,7-diBr-BTBT S-oxides_Molecules_final - ESI.pdf]

Supplementary Materials

Modulation of Properties in [1]Benzothieno[3,2-*b*][1]benzothiophene Derivatives through Sulfur Oxidation

Aneta Rzewnicka [1], Rafał Dolot [2], Maciej Mikina [1], Jerzy Krysiak [1] and Remigiusz Żurawiński [1,*]

1 Division of Organic Chemistry, Centre of Molecular and Macromolecular Studies, Polish Academy of Sciences, Sienkiewicza 112, 90-363 Lodz, Poland; aneta.rzewnicka@cbmm.lodz.pl (A.R.); maciej.mikina@cbmm.lodz.pl (M.M.); jerzy.krysiak@cbmm.lodz.pl (J.K.); remigiusz.zurawinski@cbmm.lodz.pl (R.Ż.)

2 Division of Bioorganic Chemistry, Centre of Molecular and Macromolecular Studies, Polish Academy of Sciences, Sienkiewicza 112, 90-363 Lodz, Poland; rafal.dolot@cbmm.lodz.pl (R.D.)

* Correspondence: remigiusz.zurawinski@cbmm.lodz.pl (R.Ż.)

Table of Contents:

1. NMR spectra……………………………………………………………………..S2-S3
2. X-Ray analysis…………………………………………………………………….S4-S12
3. TGA and DSC analysis…………………………………………………………......S13
4. Theorethical calculations………………………………………………………….S14-S20

Section S1. NMR spectra

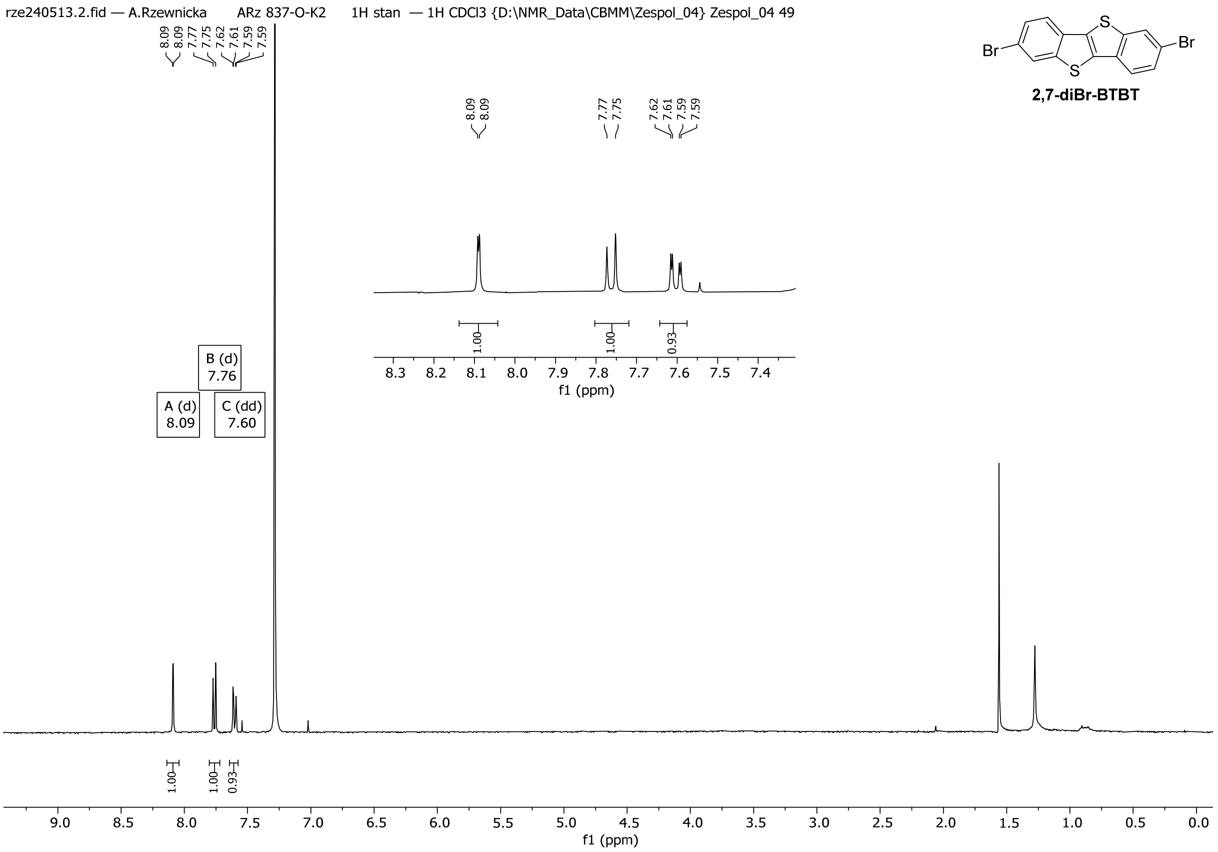

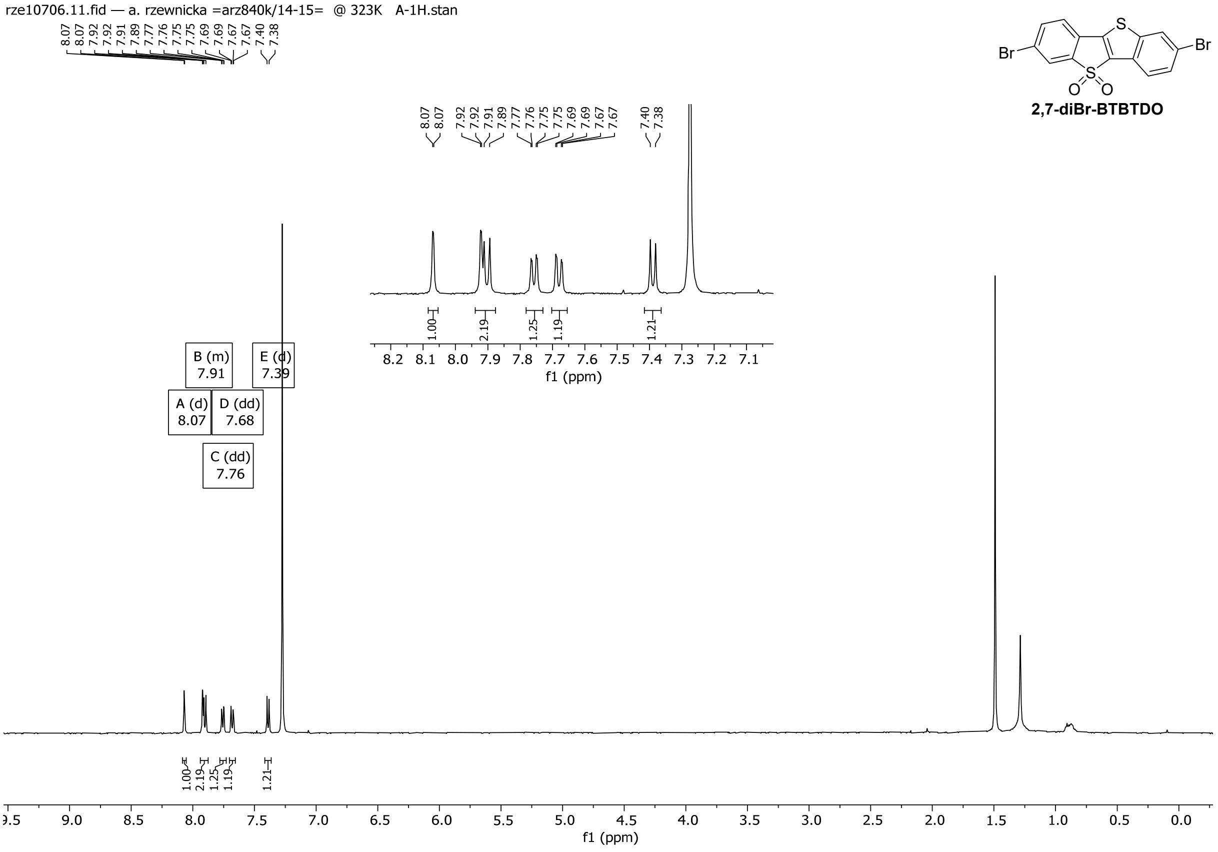

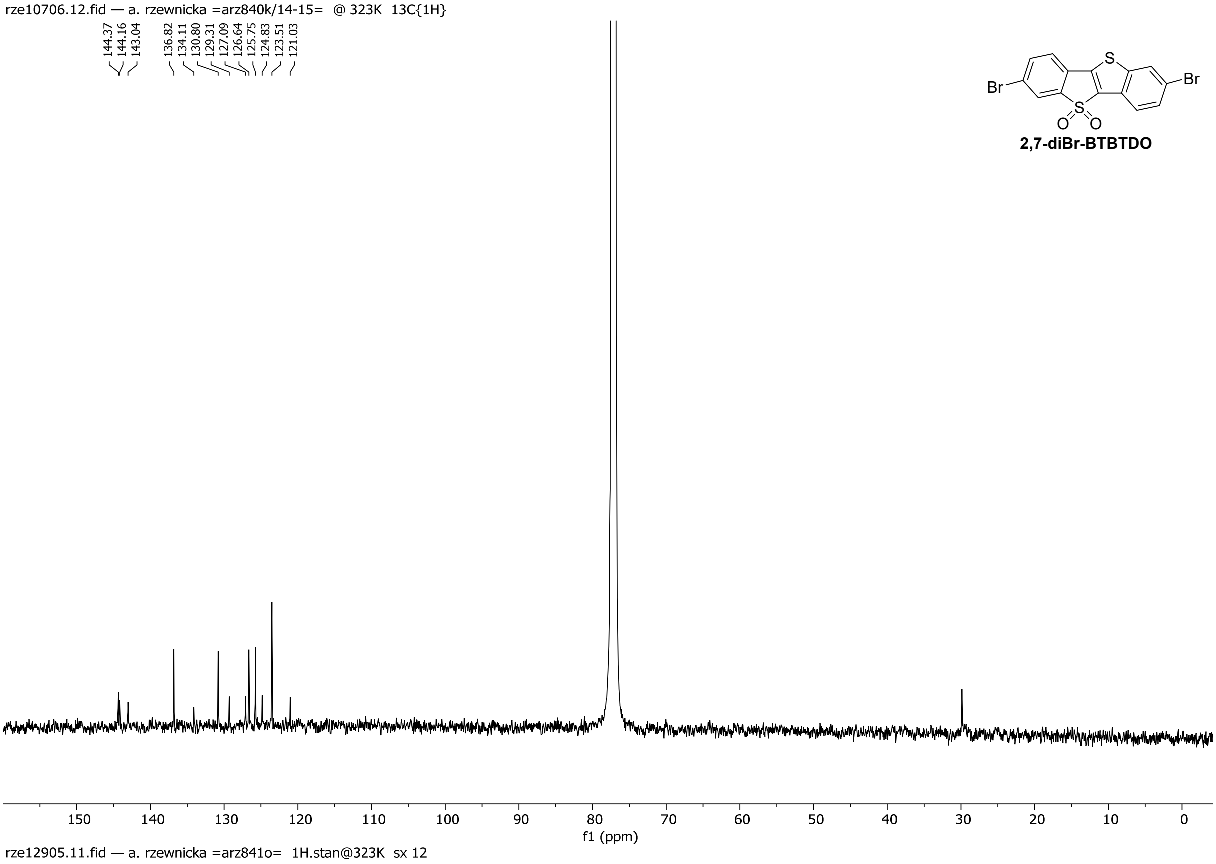
rze10706.12.fid — a. rzewnicka =arz840k/14-15= @ 323K 13C{1H}
144.37
144.16
143.04
136.82
134.11
130.80
129.31
127.09
126.64
125.75
124.83
123.51
121.03
Br
S
Br
S
O
O
2,7-diBr-BTBTDO
150
140
130
120
110
100
90
80
70
60
50
40
30
20
10
0
f1 (ppm)

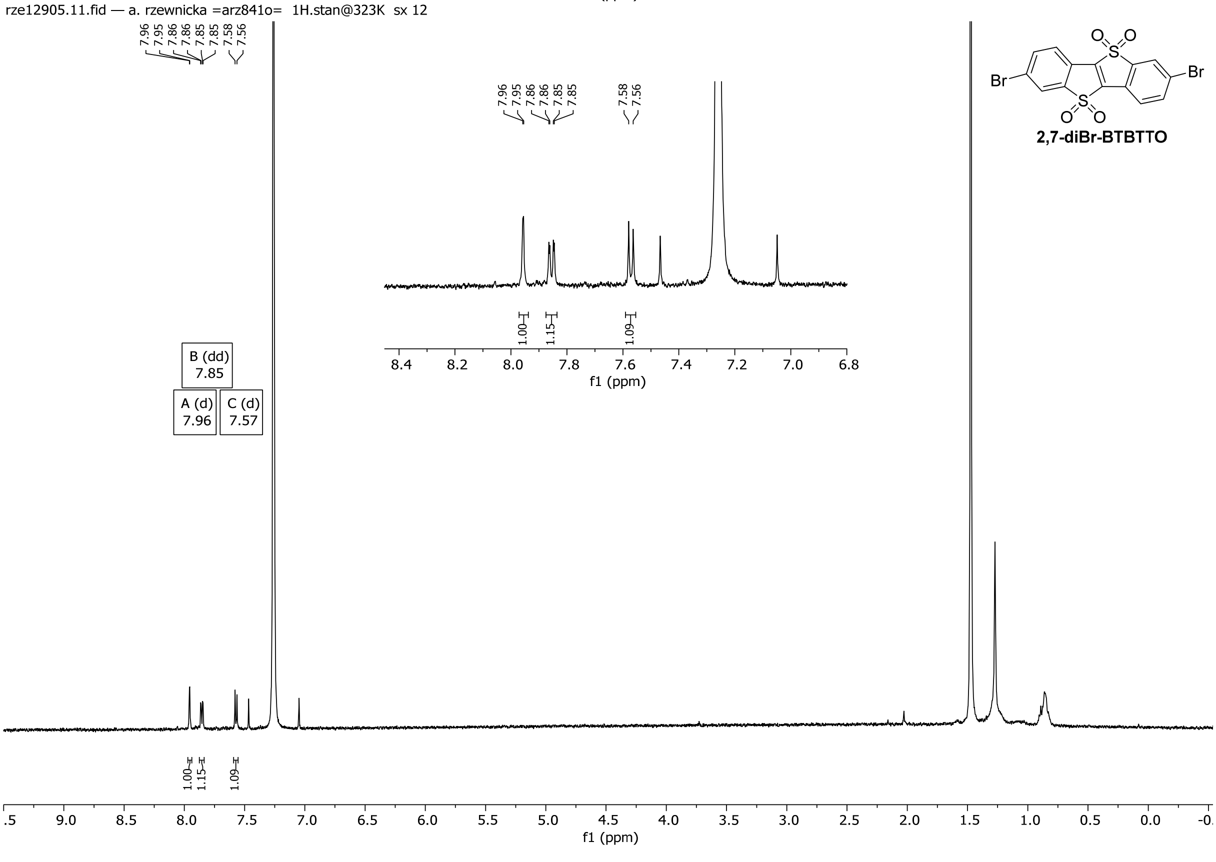
rze12905.11.fid — a. rzewnicka =arz841o= 1H.stan@323K sx 12
7.96
7.95
7.86
7.86
7.85
7.85
7.58
7.56
O
O
S
Br
Br
S
O
O
2,7-diBr-BTBTTO
B (dd)
7.85
A (d)
7.96
C (d)
7.57
1.00
1.15
1.09
8.4
8.2
8.0
7.8
7.6
7.4
7.2
7.0
6.8
f1 (ppm)
.5
9.0
8.5
8.0
7.5
7.0
6.5
6.0
5.5
5.0
4.5
4.0
3.5
3.0
2.5
2.0
1.5
1.0
0.5
0.0
-0.
f1 (ppm)

Section S2. X-Ray analysis

Table S1. Crystal structure, data collection and refinement parameters of the **2,7-diBr-BTBT** *S*-oxides studied in this research.

Compound	2,7-diBr-BTBTDO	2,7-diBr-BTBTTO
**Crystal data**		
CCDC	2368892	2368896
Chemical formula	$C_{14}H_8Br_2O_2S_2$	$C_{14}H_8Br_2O_4S_2$
Formula weight	430.13	462.13
Crystal system	triclinic	Monoclinic
Space group	*P*-1	$P2_1/c$
Temperature (K)	100.00(10)	99.95(11)
a [Å]	7.92800(10)	8.0188(4)
b [Å]	8.26170(10)	13.3257(6)
c [Å]	10.9830(2)	6.9099(3)
α [°]	110.1170(10)	90
β [°]	94.1940(10)	104.116(5)
γ [°]	93.2470(10)	90
V [Å^3]	671.102(17)	711.23(6)
**Z**	2	2
**Z'**	1	0.5
d_{calc} [g/cm^3]	2.129	2.158
Crystal dimensions [mm]	0.20 × 0.03 × 0.03	0.15 × 0.10 × 0.03
Radiation type	CuKα	CuKα
μ [mm^{-1}]	10.553	10.128
**Data collection**		
Reflections measured	24525	8599
Range/indices (*h*, *k*, *l*)	-10, 9<br>-10, 9<br>-13, 13	-10, 10<br>-16, 16<br>-8, 8
θ (max, min) [°]	77.021, 4.308	76.356, 5.689
Total no. of unique data	2656	1409
No. of observed data, I > 2σ(I)	2464	1262
R_{int}	0.059	0.062
**Refinement**		
R [$F^2 > 2\sigma(F^2)$]	0.025	0.089
$wR(F^2)$	0.071	0.251
S	1.052	1.108
No. of reflections	2656	1170
No. of parameters	182	100
No. of restraints	0	0
H-atom treatment	H atoms treated by constrained refinement	H atoms treated by constrained refinement
Δρ (min, max), e/Å^3	-0.57/0.62	-0.98/4.87

Table S2.1. Fractional atomic coordinates (×10^4) and equivalent isotropic displacement parameters (Å^2×10^3) for **2,7-diBr-BTBTDO**.

**Atom**	*x*	*y*	*z*	**U(eq)**
Br1	7656.7(3)	984.1(3)	10458.1(2)	17.17(10)
Br2	1738.9(3)	4032.9(3)	553.8(2)	17.73(10)
S5	6244.2(7)	2409.2(7)	4076.5(5)	14.42(14)
S10	3033.5(7)	2605.8(7)	6959.7(6)	14.47(14)
O11	2620(2)	4301(2)	7739.0(16)	20.2(4)
O12	1775.4(19)	1176(2)	6725.9(17)	21.6(4)
C1	5421(3)	1848(3)	8674(2)	15.2(5)
C2	7060(3)	1384(3)	8892(2)	14.8(5)
C3	8250(3)	1209(3)	7996(2)	16.1(5)
C4	7818(3)	1486(3)	6831(2)	15.2(5)
C6	3919(3)	3242(3)	2382(2)	15.5(5)
C7	2279(3)	3605(3)	2115(2)	15.3(5)
C8	1007(3)	3663(3)	2956(2)	16.7(5)
C9	1389(3)	3352(3)	4099(2)	16.6(5)
C10	6212(3)	1956(3)	6593(2)	13.9(5)
C11	5438(3)	2300(3)	5466(2)	13.9(5)
C12	4283(3)	2926(3)	3535(2)	14.3(5)
C13	3038(3)	2990(3)	4409(2)	14.2(5)
C14	3774(3)	2634(3)	5503(2)	14.5(5)
C15	5038(3)	2115(3)	7523(2)	13.8(5)
H1	4616.58	1971.14	9292.62	18
H3	9353.14	901.48	8175.59	19
H4	8618.75	1353.98	6208.56	18
H6	4760.36	3208.88	1802.82	19
H8	-106.4	3913.17	2738.96	20
H9	539.59	3384.24	4671.39	20

Table S2.2. Anisotropic displacement parameters (Å^2×10^3) for **2,7-diBr-BTBTDO**.

Atom	U_{11}	U_{22}	U_{33}	U_{23}	U_{13}	U_{12}
Br1	17.22(15)	19.73(16)	16.28(16)	8.50(11)	0.34(10)	3.08(10)
Br2	21.22(16)	17.02(15)	15.51(16)	6.68(11)	-1.26(10)	3.77(10)
S5	11.9(3)	17.5(3)	14.4(3)	5.5(2)	2.6(2)	3.9(2)
S10	10.9(3)	18.5(3)	15.8(3)	7.3(2)	3.0(2)	4.6(2)
O11	20.6(8)	23.4(9)	18.7(9)	7.8(7)	6.5(7)	10.5(7)
O12	12.6(8)	25.0(9)	29.0(10)	11.5(8)	3.0(7)	1.8(7)
C1	16.5(11)	13.9(11)	16.0(12)	6.1(10)	1.8(9)	2.4(9)
C2	15.7(11)	11.8(10)	15.7(11)	3.7(9)	-0.1(9)	1.1(8)
C3	15.0(11)	13.6(11)	19.4(12)	5.7(10)	-1.2(9)	1.9(9)
C4	13.3(11)	15.0(11)	17.0(12)	4.9(9)	3.4(9)	2.0(8)
C6	18.8(12)	13.2(11)	14.8(12)	4.8(9)	2.3(9)	3.7(9)
C7	20.3(12)	10.7(10)	12.9(11)	1.6(9)	-0.3(9)	2.9(9)
C8	15.3(11)	15.1(11)	17.3(12)	3.1(9)	-2.0(9)	3.6(9)
C9	14.2(11)	18.1(11)	16.8(12)	4.7(9)	2.2(9)	2.7(9)
C10	14.2(11)	13.3(11)	12.8(11)	2.9(9)	0.9(9)	0.3(8)
C11	14.3(11)	12.4(10)	14.7(11)	4.3(9)	1.8(9)	1.4(8)
C12	13.3(11)	13.4(11)	15.2(11)	3.6(9)	1.4(9)	3.1(8)
C13	15.2(11)	13.0(11)	12.5(11)	2.0(9)	1.1(9)	1.9(8)
C14	13.8(11)	13.7(11)	15.3(11)	3.7(9)	2.2(9)	2.1(8)
C15	11.0(10)	13.6(11)	17.5(12)	6.0(9)	2.0(9)	3.5(8)

Table S2.3. Bond lengths for **2,7-diBr-BTBTDO** (Å).

Atom – Atom	Length	Atom – Atom	Length
Br1 — C2	1.892(2)	C8 — C9	1.381(3)
Br2 — C7	1.891(2)	C8 — C7	1.409(3)
S10 — O12	1.4412(17)	C13 — C9	1.401(3)
S10 — O11	1.4386(17)	C13 — C12	1.418(3)
S10 — C15	1.782(2)	C13 — C14	1.425(3)
S10 — C14	1.751(2)	C4 — C10	1.384(3)
S5 — C11	1.725(2)	C11 — C14	1.364(3)
S5 — C12	1.751(2)	C11 — C10	1.463(3)
C3 — C4	1.398(3)	C15 — C10	1.410(3)

Atom – Atom	Length	Atom – Atom	Length
C3 — C2	1.390(3)	C6 — C12	1.392(3)
C1 — C15	1.372(3)	C6 —C7	1.384(3)
C1 — C2	1.402(3)		

Table S2.4. Bond angles for **2,7-diBr-BTBTDO** (°).

Atom – Atom – Atom	Angle	Atom – Atom – Atom	Angle
O12 — S10 — C15	110.29(10)	C10 — C15 — S10	110.89(18)
O12 — S10 — C14	111.25(11)	C8 — C9 — C13	119.6(2)
O11 — S10 — O12	117.67(10)	C7 — C6 — C12	117.2(2)
O11 — S10 — C15	111.89(11)	C13 — C12 — S5	112.55(18)
O11 — S10 — C14	110.61(11)	C6 — C12 — S5	125.81(17)
C14 — S10 — C15	92.29(11)	C6 — C12 — C13	121.6(2)
C11 — S5 — C12	90.80(11)	C13 — C14 — S10	133.97(17)
C2 — C3 — C4	119.8(2)	C11 — C14 — S10	110.71(19)
C15 — C1 — C2	116.7(2)	C11 — C14 — C13	115.3(2)
C9 — C8 — C7	119.5(2)	C4 — C10 — C11	130.4(2)
C9 — C13 — C12	119.4(2)	C4 — C10 — C15	119.2(2)
C9 — C13 — C14	131.4(2)	C15 — C10 — C11	110.4(2)
C12 — C13 — C14	109.2(2)	C8 — C7 — Br2	119.12(18)
C10 — C4 — C3	119.4(2)	C6 — C7 — Br2	118.27(17)
C14 — C11 — S5	112.2(2)	C6 — C7 — C8	122.6(2)
C14 — C11 — C10	115.7(2)	C3 — C2 — Br1	119.29(18)
C10 — C11 — S5	132.10(17)	C3 — C2 — C1	122.1(2)
C1 — C15 — S10	126.24(17)	C1 — C2 — Br1	118.62(17)
C1 — C15 — C10	122.8(2)		

Table S2.5. Torsion angles for **2,7-diBr-BTBTDO** (°).

Atom – Atom – Atom – Atom	Angle	Atom – Atom – Atom – Atom	Angle
S10 — C15 — C10 — C4	-176.65(17)	C9 — C8 — C7 — C6	0.2(4)
S10 — C15 — C10 — C11	1.9(2)	C9 — C13 — C12 — S5	-178.67(17)
S5 — C11 — C14 — S10	-177.34(10)	C9 — C13 — C12 — C6	1.1(3)
S5 — C11 — C14 — C13	0.3(3)	C9 — C13 — C14 — S10	-4.3(4)
S5 — C11 — C10 — C4	-5.3(4)	C9 — C13 — C14 — C11	178.7(2)

Atom – Atom – Atom – Atom	Angle	Atom – Atom – Atom – Atom	Angle
S5 — C11 — C10 — C15	176.36(19)	C12 — S5 — C11 — C14	0.2(2)
O12 — S10 — C15 — C1	-64.8(2)	C12 — S5 — C11 — C10	-178.5(2)
O12 — S10 — C15 — C10	112.75(17)	C12 — C13 — C9 — C8	-0.8(3)
O12 — S10 — C14 — C13	69.8(3)	C12 — C13 — C14 — S10	176.16(19)
O12 — S10 — C14 — C11	-113.18(18)	C12 — C13 — C14 — C11	-0.8(3)
O11 — S10 — C15 — C1	68.2(2)	C12 — C6 — C7 — Br2	-179.79(16)
O11 — S10 — C15 — C10	-114.24(17)	C12 — C6 — C7 — C8	0.0(3)
O11 — S10 — C14 — C13	-63.0(3)	C14 — S10 — C15 — C1	-178.4(2)
O11 — S10 — C14 — C11	114.08(19)	C14 — S10 — C15 — C10	-0.93(18)
C3 — C4 — C10 — C11	-179.3(2)	C14 — C13 — C9 — C8	179.7(2)
C3 — C4 — C10 — C15	-1.1(3)	C14 — C13 — C12 — S5	0.9(2)
C1 — C15 — C10 — C4	1.0(3)	C14 — C13 — C12 — C6	-179.4(2)
C1 — C15 — C10 — C11	179.5(2)	C14 — C11 — C10 — C4	176.0(2)
C4 — C3 — C2 — Br1	179.46(17)	C14 — C11 — C10 — C15	-2.3(3)
C4 — C3 — C2 — C1	-0.5(3)	C10 — C11 — C14 — S10	1.6(3)
C11 — S5 — C12 — C13	-0.65(18)	C10 — C11 — C14 — C13	179.20(18)
C11 — S5 — C12 — C6	179.6(2)	C7 — C8 — C9 — C13	0.2(3)
C15 — S10 — C14 — C13	-177.4(2)	C7 — C6 — C12 — S5	179.04(17)
C15 — S10 — C14 — C11	-0.4(2)	C7 — C6 — C12 — C13	-0.6(3)
C15 — C1 — C2 — Br1	-179.61(16)	C2 — C3 — C4 — C10	0.9(3)
C15 — C1 — C2 — C3	0.4(3)	C2 — C1 — C15 — S10	176.64(17)
C9 — C8 — C7 — Br2	-179.99(17)	C2 — C1 — C15 — C10	-0.6(3)

Table S3.1. Fractional atomic coordinates (×10^4) and equivalent isotropic displacement parameters (Å^2×10^3) for **2,7-diBr-BTBTTO**.

Atom	*x*	*y*	*z*	U(eq)
Br1	2268.4(11)	9507.2(6)	4225.2(12)	24.1(5)
S8	3194(2)	3822.9(14)	4464(3)	17.6(5)
O9	2270(9)	3648(5)	5967(11)	33.4(15)
O10	2343(9)	3632(5)	2402(10)	32.3(15)
C1	4130(10)	5035(6)	4747(11)	17.1(15)
C2	3429(10)	6055(6)	4530(10)	14.5(14)
C3	1729(11)	6380(6)	4059(11)	19.8(16)

Atom	*x*	*y*	*z*	U(eq)
C4	1400(10)	7422(6)	3971(11)	20.7(16)
C5	2759(11)	8121(6)	4351(11)	19.6(15)
C6	4459(11)	7807(6)	4830(11)	19.6(16)
C7	4778(10)	6769(6)	4906(11)	17.6(15)
H3	810.84	5906.95	3802.12	24
H4	246.36	7657.69	3651.44	25
H6	5375.88	8282.01	5096.65	24

Table S3.2. Anisotropic displacement parameters (Å^2×10^3) for **2,7-diBr-BTBTTO**.

Atom	U_{11}	U_{22}	U_{33}	U_{23}	U_{13}	U_{12}
Br1	36.4(7)	15.8(6)	22.0(6)	1.2(3)	10.5(4)	5.1(3)
S8	19.3(9)	14.9(9)	20.1(10)	-0.3(6)	7.6(7)	-0.9(6)
O10	33(3)	31(3)	31(3)	-4(3)	3(3)	-5(3)
O9	32(3)	33(4)	40(4)	1(3)	18(3)	-2(3)
C2	21(3)	13(3)	13(3)	0(2)	11(3)	-3(3)
C3	23(4)	23(4)	16(3)	2(3)	10(3)	2(3)
C1	24(4)	18(4)	12(3)	0(3)	10(3)	-3(3)
C4	22(4)	21(4)	20(3)	4(3)	6(3)	5(3)
C5	31(4)	17(4)	14(4)	0(3)	12(3)	4(3)
C6	32(4)	13(4)	16(3)	-1(3)	12(3)	-9(3)
C7	23(4)	17(4)	14(3)	-1(2)	7(3)	-1(3)

Table S3.3. Bond lengths for **2,7-diBr-BTBTTO** (Å).

Atom – Atom	Length	Atom – Atom	Length
Br1 — C5	1.874(8)	C2 — C7	1.411(10)
S8 — O10	1.444(7)	C3 — C4	1.403(12)
S8 — O9	1.435(7)	C1 — C1[1]	1.356(16)
S8 — C1	1.762(8)	C4 — C5	1.404(12)
S8 — C7[1]	1.761(8)	C5 — C6	1.386(12)
C2 — C3	1.390(11)	C6 — C7	1.397(11)
C2 — C1	1.456(11)		

[1] Symmetry code:1-X,1-Y,1-Z

Table S3.4. Bond angles for **2,7-diBr-BTBTTO** (°).

**Atom – Atom – Atom**	**Angle**	**Atom – Atom – Atom**	**Angle**
O10 — S8 — C1	110.8(4)	C1[1] — C1 — S8	110.5(8)
O10 — S8 — C7[1]	110.6(4)	C1[1] — C1 — C2	115.9(9)
O9 — S8 — O10	118.9(4)	C3 — C4 — C5	120.7(8)
O9 — S8 — C1	110.3(4)	C4 — C5 — Br1	119.4(6)
O9 — S8 — C7[1]	111.0(4)	C6 — C5 — Br1	119.2(6)
C7[1] — S8 — C1	92.0(4)	C6 — C5 — C4	121.4(7)
C3 — C2 — C1	130.0(7)	C5 — C6 — C7	117.6(7)
C3 — C2 — C7	120.0(7)	C2 — C7 — S8[1]	111.6(6)
C7 — C2 — C1	110.0(7)	C6 — C7 — S8[1]	126.6(6)
C2 — C3 — C4	118.5(8)	C6 — C7 — C2	121.8(7)
C2 — C1 — S8	133.6(6)		

[1] Symmetry code:1-X,1-Y,1-Z

Table S3.5. Torsion angles for **2,7-diBr-BTBTTO** (°).

**Atom – Atom – Atom – Atom**	**Angle**	**Atom – Atom – Atom – Atom**	**Angle**
Br1 — C5 — C6 — C7	-179.9(6)	C1 — C2 — C3 — C4	179.1(7)
O10 — S8 — C1 — C2	67.3(8)	C1 — C2 — C7 — S8[1]	-1.0(8)
O10 — S8 — C1 — C1[1]	-111.9(8)	C1 — C2 — C7 — C6	-178.9(7)
O9 — S8 — C1 — C2	-66.6(8)	C4 — C5 — C6 — C7	0.5(11)
O9 — S8 — C1 — C1[1]	114.3(8)	C5 — C6 — C7 — S8[1]	-178.2(5)
C2 — C3 — C4 — C5	0.0(11)	C5 — C6 — C7 — C2	-0.6(11)
C3 — C2 — C1 — S8	1.9(13)	C7[1] — S8 — C1 — C2	-179.8(7)
C3 — C2 — C1 — C1[1]	-179.0(9)	C7[1] — S8 — C1 — C1[1]	1.1(8)
C3 — C2 — C7 — S8[1]	178.3(6)	C7 — C2 — C3 — C4	-0.1(11)
C3 — C2 — C7 — C6	0.4(12)	C7 — C2 — C1 — S8	-178.9(6)
C3 — C4 — C5 — Br1	-179.9(5)	C7 — C2 — C1 — C1[1]	0.2(11)
C3 — C4 — C5 — C6	-0.2(11)		

[1] Symmetry code:1-X,1-Y,1-Z

Table S4.1. List of intermolecular short contacts in the crystal structure of **2,7-diBr-BTBT**.

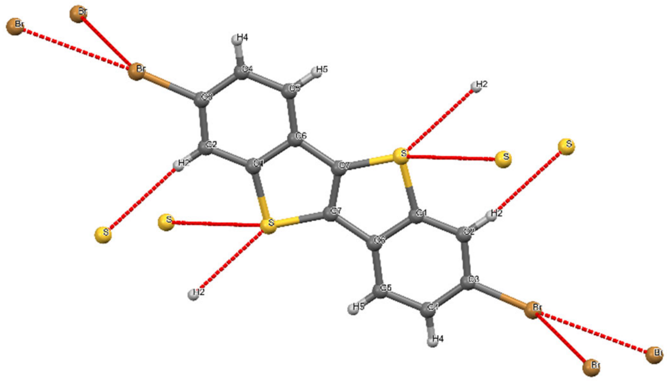

Number	Atom1	Atom2	Atom1 – Atom2 distance [Å]
1	S1	S1	3.533
2	S1	S1	3.533
3	S1	H2	2.901
4	S1	H2	2.901
5	H2	S1	2.901
6	H2	S1	2.901
7	Br1	Br1	3.587
8	Br1	Br1	3.587
9	Br1	Br1	3.587
10	Br1	Br1	3.587

Table S4.2. List of intermolecular short contacts in the crystal structure of **2,7-diBr-BTBTDO**.

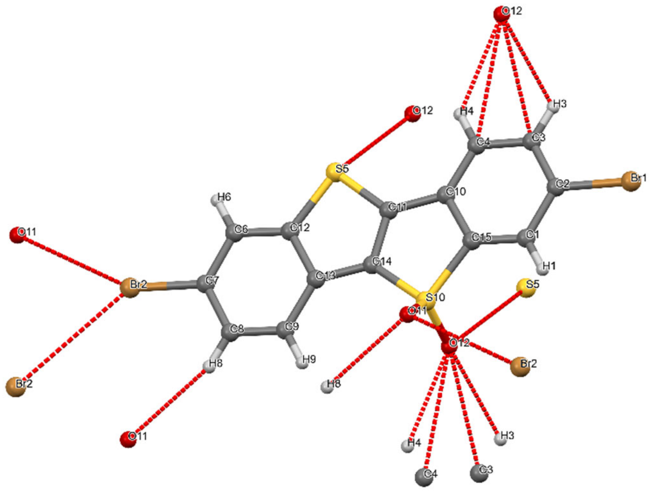

Number	Atom1	Atom2	Atom1 – Atom2 distance [Å]
1	S5	O12	3.309
2	O12	S5	3.309
3	Br2	Br2	3.903
4	Br2	O11	3.298
5	O11	Br2	3.298
6	O12	C3	3.211
7	C3	O12	3.211
8	O12	C4	3.170
9	C4	O12	3.170
10	O12	H3	2.631
11	H3	O12	2.631
12	O12	H4	2.649
13	H4	O12	2.649
14	O11	H8	2.871
15	H8	O11	2.871

Table S4.3. List of intermolecular short contacts in the crystal structure of **2,7-diBr-BTBTTO**.

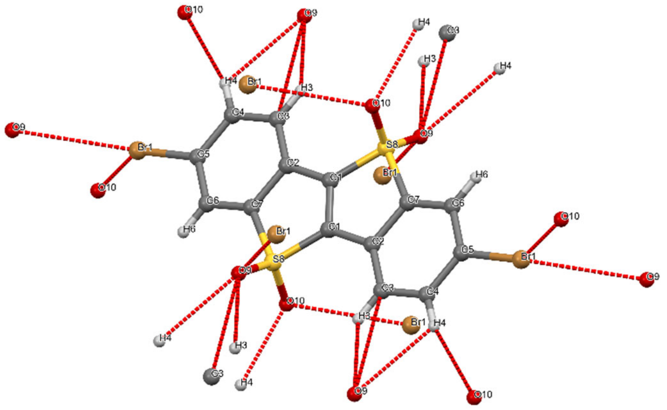

Number	Atom1	Atom2	Atom1 – Atom2 distance [Å]
1	Br1	O9	3.321
2	Br1	O9	3.321
3	O9	Br1	3.321
4	O9	Br1	3.321
5	Br1	O10	3.290
6	Br1	O10	3.290
7	O10	Br1	3.290
8	O10	Br1	3.290
9	O9	C3	3.203
10	O9	C3	3.203
11	C3	O9	3.203
12	C3	O9	3.203
13	O9	H3	2.582
14	O9	H3	2.582
15	H3	O9	2.582
16	H3	O9	2.582
17	O9	H4	2.718
18	O9	H4	2.718
19	H4	O9	2.718
20	H4	O9	2.718
21	O10	H4	2.401
22	O10	H4	2.401
23	H4	O10	2.401
24	H4	O10	2.401

Section S3. TGA and DSC analysis

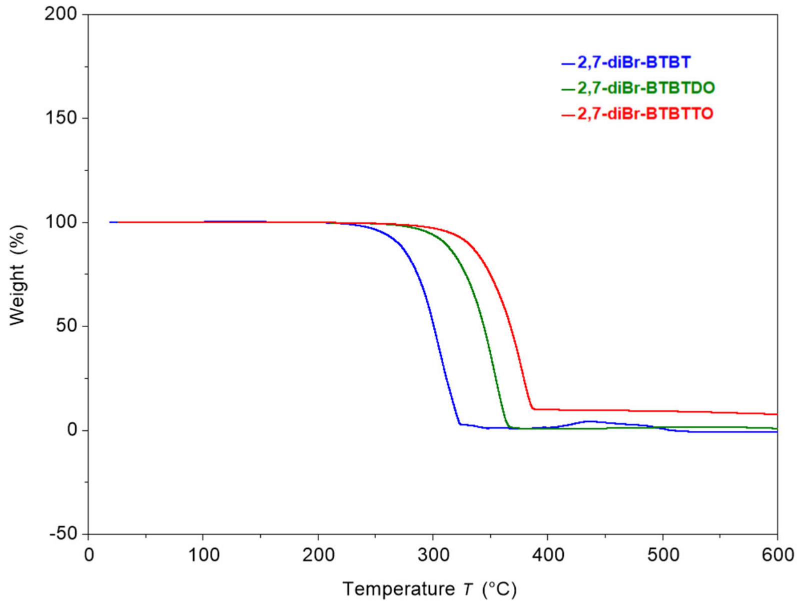

Figure S1. TGA analysis for **2,7-diBr-BTBT** *S*-oxides.

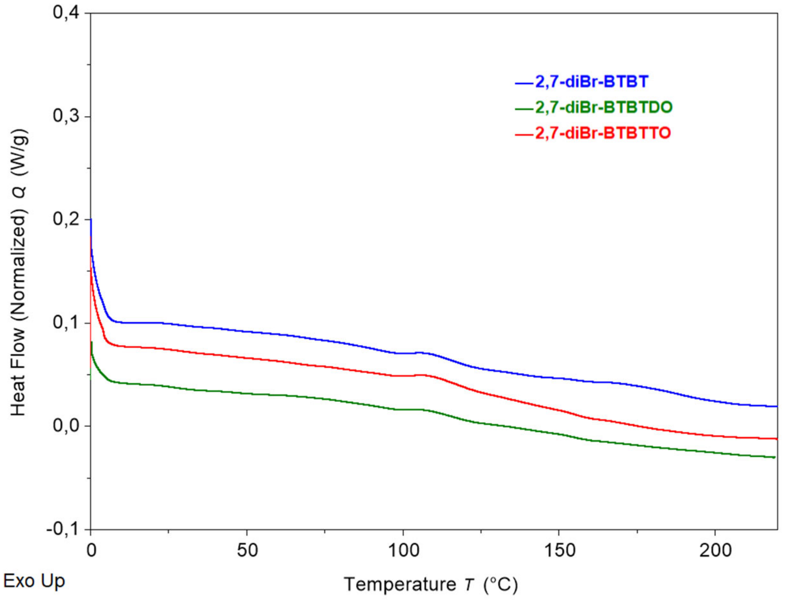

Figure S2. DSC analysis for **2,7-diBr-BTBT** *S*-oxides.

Section S4. Theorethical calculations

Atomic coordinates of **2,7-diBr-BTBT** and its *S*-oxides optimized at the TDDFT PBE0/6-311+G(2d,p)//D3-M06-2X/def2-TZVP level in DCM (IEFPCM solvation model):

2,7-diBr-BTBT

0 1

C	-0.65728575	2.20447858	0.00000000
C	0.65728575	1.69622265	0.00000000
C	0.63062642	0.26417244	0.00000000
C	-0.63062642	-0.26417244	0.00000000
C	0.65728575	-2.20447858	0.00000000
C	-0.65728575	-1.69622265	0.00000000
S	-1.86729842	0.94915675	0.00000000
S	1.86729842	-0.94915675	0.00000000
C	-1.49990041	-3.94402104	0.00000000
H	-2.32342867	-4.64370684	0.00000000
C	-1.73563480	-2.58543170	0.00000000
H	-2.75134930	-2.21100449	0.00000000
C	0.90607331	-3.57398650	0.00000000
H	1.91447817	-3.96359843	0.00000000
C	-0.90607331	3.57398650	0.00000000
H	-1.91447817	3.96359843	0.00000000
C	1.49990041	3.94402104	0.00000000
H	2.32342867	4.64370684	0.00000000
C	1.73563480	2.58543170	0.00000000
H	2.75134930	2.21100449	0.00000000
C	-0.18462920	-4.41850990	0.00000000
C	0.18462920	4.41850990	0.00000000
Br	0.10807843	-6.29275269	0.00000000
Br	-0.10807843	6.29275269	0.00000000

2,7-diBr-BTBTDO

0 1

C	-2.29397127	-0.81634170	-0.00084654
C	-1.79462196	0.50243765	0.00007307
C	-0.36892373	0.47040246	0.00091463
C	0.16812703	-0.77459662	0.00089247
C	2.18164188	0.44602582	0.00036364
C	1.63064345	-0.83565131	0.00184994
S	-1.02390912	-2.00988982	-0.00104827
S	0.89968754	1.68645123	0.00079152
O	0.91381893	2.42756747	1.23261273
O	0.91267354	2.42836323	-1.23051724
C	2.47288547	-1.93339092	0.00441145
H	2.07507951	-2.93975964	0.00707195
C	3.53515926	0.68141363	-0.00039706
H	3.94278185	1.68335206	-0.00181731
C	-3.66131872	-1.07861032	-0.00148515
H	-4.04275776	-2.08995443	-0.00243745
C	-4.04390619	1.32667493	0.00025722

H	-4.75081002	2.14390692	0.00086672
C	-2.68989881	1.57760590	0.00049158
H	-2.32322180	2.59558276	0.00138015
C	4.36343019	-0.43902763	0.00044381
C	-4.51107666	0.00676151	-0.00059748
C	3.84890076	-1.72743048	0.00343566
H	4.52152493	-2.57337240	0.00505482
Br	6.23689687	-0.18966215	-0.00186603
Br	-6.38173734	-0.29404702	-0.00046592

2,7-diBr-BTBTTO

0 1

C	0.14050915	2.35913519	0.00000000
C	-1.00674486	1.56368592	0.00000000
C	-0.64927591	0.14889710	0.00000000
C	0.64927591	-0.14889710	0.00000000
C	-0.14050915	-2.35913519	0.00000000
C	1.00674486	-1.56368592	0.00000000
S	1.60903885	1.34802953	0.00000000
O	2.32464586	1.47868651	1.23546534
O	2.32464586	1.47868651	-1.23546534
S	-1.60903885	-1.34802953	0.00000000
O	-2.32464586	-1.47868651	-1.23546534
O	-2.32464586	-1.47868651	1.23546534
C	2.25270005	-2.16409350	0.00000000
H	3.15787849	-1.57168885	0.00000000
C	-0.10046771	-3.73106802	0.00000000
H	-0.99961881	-4.33211612	0.00000000
C	0.10046771	3.73106802	0.00000000
H	0.99961881	4.33211612	0.00000000
C	-2.32464586	3.55393626	0.00000000
H	-3.28831622	4.04313204	0.00000000
C	-2.25270005	2.16409350	0.00000000
H	-3.15787849	1.57168885	0.00000000
C	1.16580682	-4.31642016	0.00000000
C	-1.16580682	4.31642016	0.00000000
C	2.32464586	-3.55393626	0.00000000
H	3.28831622	-4.04313204	0.00000000
Br	1.29738178	-6.19941366	0.00000000
Br	-1.29738178	6.19941366	0.00000000

Atomic coordinates of **2,7-diBr-BTBT** and its *S*-oxides optimized at the D3-M06-2X/def2-TZVPD:

2,7-diBr-BTBT (neutral)

0 1

C	-0.65691983	2.20468596	0.00000000
C	0.65691983	1.69579562	0.00000000
C	0.63035428	0.26436007	0.00000000
C	-0.63035428	-0.26436007	0.00000000
C	0.65691983	-2.20468596	0.00000000
C	-0.65691983	-1.69579562	0.00000000

S	-1.86628722	0.94890532	0.00000000
S	1.86628722	-0.94890532	0.00000000
C	-1.49900261	-3.94376472	0.00000000
H	-2.32111279	-4.64507385	0.00000000
C	-1.73414575	-2.58549095	0.00000000
H	-2.74948779	-2.20936762	0.00000000
C	0.90412337	-3.57394793	0.00000000
H	1.91167982	-3.96586823	0.00000000
C	-0.90412337	3.57394793	0.00000000
H	-1.91167982	3.96586823	0.00000000
C	1.49900261	3.94376472	0.00000000
H	2.32111279	4.64507385	0.00000000
C	1.73414575	2.58549095	0.00000000
H	2.74948779	2.20936762	0.00000000
C	-0.18475367	-4.42093504	0.00000000
C	0.18475367	4.42093504	0.00000000
Br	0.10740252	-6.29177879	0.00000000
Br	-0.10740252	6.29177879	0.00000000

2,7-diBr-BTBT (cation)

1 2

C	-0.57574505	2.21095745	0.00000000
C	0.72730624	1.66059201	0.00000000
C	0.66623530	0.25108368	0.00000000
C	-0.66623530	-0.25108368	0.00000000
C	0.57574505	-2.21095745	0.00000000
C	-0.72730624	-1.66059201	0.00000000
S	-1.83896739	0.98145453	0.00000000
S	1.83896739	-0.98145453	0.00000000
C	-1.64232072	-3.88046064	0.00000000
H	-2.48325183	-4.55897737	0.00000000
C	-1.83896739	-2.51904526	0.00000000
H	-2.84247476	-2.11233340	0.00000000
C	0.78785476	-3.56852053	0.00000000
H	1.78084044	-3.99659683	0.00000000
C	-0.78785476	3.56852053	0.00000000
H	-1.78084044	3.99659683	0.00000000
C	1.64232072	3.88046064	0.00000000
H	2.48325183	4.55897737	0.00000000
C	1.83896739	2.51904526	0.00000000
H	2.84247476	2.11233340	0.00000000
C	-0.34043368	-4.39695364	0.00000000
C	0.34043368	4.39695364	0.00000000
Br	-0.09267422	-6.24563729	0.00000000
Br	0.09267422	6.24563729	0.00000000

2,7-diBr-BTBT (anion)

-1 2

C	-0.64143955	2.22501665	0.00000000
C	0.68273017	1.67705424	0.00000000
C	0.64143955	0.28297954	0.00000000

C	-0.64143955	-0.28297954	0.00000000
C	0.64143955	-2.22501665	0.00000000
C	-0.68273017	-1.67705424	0.00000000
S	-1.86800114	0.96303936	0.00000000
S	1.86800114	-0.96303936	0.00000000
C	-1.53252839	-3.95031601	0.00000000
H	-2.36250512	-4.64485878	0.00000000
C	-1.76979926	-2.58865709	0.00000000
H	-2.78625923	-2.21583880	0.00000000
C	0.87629906	-3.57951040	0.00000000
H	1.88269170	-3.97603273	0.00000000
C	-0.87629906	3.57951040	0.00000000
H	-1.88269170	3.97603273	0.00000000
C	1.53252839	3.95031601	0.00000000
H	2.36250512	4.64485878	0.00000000
C	1.76979926	2.58865709	0.00000000
H	2.78625923	2.21583880	0.00000000
C	-0.23060292	-4.44050070	0.00000000
C	0.23060292	4.44050070	0.00000000
Br	0.06649705	-6.32379375	0.00000000
Br	-0.06649705	6.32379375	0.00000000

2,7-diBr-BTBTDO (neutral)

0 1

C	-2.29598455	-0.82165384	-0.00000697
C	-1.79296375	0.49499005	-0.00001528
C	-0.36810403	0.46480405	-0.00002276
C	0.16936090	-0.77777912	-0.00001829
C	2.17980844	0.44624325	0.00000302
C	1.63139902	-0.83647212	-0.00000792
S	-1.02524426	-2.01573051	-0.00001517
S	0.89735408	1.69261650	-0.00000614
O	0.90790401	2.41723603	1.23600775
O	0.90794472	2.41724365	-1.23601543
C	2.47639753	-1.93266827	-0.00001335
H	2.07835441	-2.93948400	-0.00002329
C	3.53316471	0.68018138	0.00000833
H	3.93747108	1.68348052	0.00001649
C	-3.66367181	-1.07892696	0.00000162
H	-4.05204364	-2.08768991	0.00000683
C	-4.03920374	1.32573290	-0.00000570
H	-4.74565011	2.14346183	-0.00000475
C	-2.68462180	1.57246961	-0.00001514
H	-2.30896723	2.58744391	-0.00002142
C	4.36573429	-0.43702460	0.00000475
C	-4.51233639	0.00786656	0.00000283
C	3.85191421	-1.72598047	-0.00000582
H	4.52827834	-2.56917796	-0.00000854
Br	6.23706028	-0.18552121	0.00001369
Br	-6.38058447	-0.28918571	0.00001435

2,7-diBr-BTBTDO (cation)

1 2

C	2.27764070	-0.84388253	0.00000723
C	1.76972894	0.49197235	0.00001650
C	0.38478301	0.47479476	0.00002774
C	-0.17829500	-0.80724994	0.00001781
C	-2.15532334	0.46206854	-0.00000975
C	-1.60630215	-0.83741787	0.00000424
S	0.99711340	-2.04264850	0.00000768
S	-0.88476985	1.71896013	0.00000220
O	-0.87341191	2.41638032	-1.24257919
O	-0.87348044	2.41639718	1.24257480
C	-2.46338482	-1.94426252	0.00001355
H	-2.06545288	-2.95125112	0.00002744
C	-3.50190985	0.69476439	-0.00001551
H	-3.91191516	1.69620739	-0.00002477
C	3.62519921	-1.09744335	-0.00000122
H	4.02709859	-2.10130710	-0.00000728
C	4.01393890	1.34034888	0.00001082
H	4.72701379	2.15231246	0.00001352
C	2.67073893	1.58693987	0.00002024
H	2.28769316	2.59956032	0.00002891
C	-4.34048647	-0.42869235	-0.00001018
C	4.48719255	0.00989193	0.00000068
C	-3.82450012	-1.73336428	0.00000697
H	-4.50856304	-2.57048154	0.00001469
Br	-6.18393908	-0.18148547	-0.00000859
Br	6.31730038	-0.29048749	-0.00001170

2,7-diBr-BTBTDO (anion)

-1 2

C	-0.43153615	-2.26163458	0.59942200
C	0.71376095	-1.67831459	-0.00858878
C	0.68733029	-0.27435253	0.12294278
C	-0.42667391	0.23209081	0.80376562
C	0.67929638	2.23157651	0.33690375
C	-0.46722373	1.63020829	0.94379683
S	-1.49147819	-1.05325271	1.30725072
S	1.74262934	1.01066537	-0.37560648
O	1.81197608	1.14601788	-1.81539848
O	3.02471077	0.96929222	0.29566450
C	-1.40440236	2.49064322	1.55263607
H	-2.28905297	2.08070503	2.02523696
C	0.89186199	3.59024433	0.32850826
H	1.77146977	4.01290761	-0.14043378
C	-0.64048173	-3.62792224	0.60618610
H	-1.51516430	-4.06177446	1.07148639
C	1.44409912	-3.90026990	-0.61042625
H	2.16264623	-4.56049064	-1.07631319
C	1.64838018	-2.53149852	-0.61457460

H	2.52841926	-2.11144481	-1.08395490
C	-0.05621904	4.39886496	0.93941624
C	0.31159946	-4.43362848	-0.00580451
C	-1.19854380	3.85139476	1.54815612
H	-1.91697266	4.51184822	2.01470232
Br	0.18487186	6.28799428	0.95819871
Br	0.05318177	-6.32205387	-0.01326068

2,7-diBr-BTBTTO (neutral)

0 1

C	0.14490795	2.35734400	0.00000000
C	-1.00274093	1.56320914	0.00000000
C	-0.64922997	0.14814209	0.00000000
C	0.64922997	-0.14814209	0.00000000
C	-0.14490795	-2.35734400	0.00000000
C	1.00274093	-1.56320914	0.00000000
S	1.61772520	1.34428205	0.00000000
O	2.32253609	1.47203523	1.23863544
O	2.32253609	1.47203523	-1.23863544
S	-1.61772520	-1.34428205	0.00000000
O	-2.32253609	-1.47203523	-1.23863544
O	-2.32253609	-1.47203523	1.23863544
C	2.24960450	-2.16291803	0.00000000
H	3.15135556	-1.56432918	0.00000000
C	-0.10135676	-3.72929748	0.00000000
H	-1.00003261	-4.33118797	0.00000000
C	0.10135676	3.72929748	0.00000000
H	1.00003261	4.33118797	0.00000000
C	-2.32253609	3.55218149	0.00000000
H	-3.28488777	4.04431554	0.00000000
C	-2.24960450	2.16291803	0.00000000
H	-3.15135556	1.56432918	0.00000000
C	1.16394493	-4.31619616	0.00000000
C	-1.16394493	4.31619616	0.00000000
C	2.32253609	-3.55218149	0.00000000
H	3.28488777	-4.04431554	0.00000000
Br	1.29570809	-6.19806367	0.00000000
Br	-1.29570809	6.19806367	0.00000000

2,7-diBr-BTBTTO (cation)

1 2

C	0.17258389	2.34187494	0.00000000
C	-0.99214921	1.53372546	0.00000000
C	-0.66663683	0.15840416	0.00000000
C	0.66663683	-0.15840416	0.00000000
C	-0.17258389	-2.34187494	0.00000000
C	0.99214921	-1.53372546	0.00000000
S	1.65424138	1.33495337	0.00000000
O	2.33480836	1.44721046	1.24473188
O	2.33480836	1.44721046	-1.24473188
S	-1.65424138	-1.33495337	0.00000000

O	-2.33480836	-1.44721046	-1.24473188
O	-2.33480836	-1.44721046	1.24473188
C	2.26044472	-2.13778056	0.00000000
H	3.15725427	-1.53097258	0.00000000
C	-0.12082239	-3.70134339	0.00000000
H	-1.01071614	-4.31712003	0.00000000
C	0.12082239	3.70134339	0.00000000
H	1.01071614	4.31712003	0.00000000
C	-2.33480836	3.51051226	0.00000000
H	-3.29454258	4.00827717	0.00000000
C	-2.26044472	2.13778056	0.00000000
H	-3.15725427	1.53097258	0.00000000
C	1.16191933	-4.28685014	0.00000000
C	-1.16191933	4.28685014	0.00000000
C	2.33480836	-3.51051226	0.00000000
H	3.29454258	-4.00827717	0.00000000
Br	1.30074825	-6.13286115	0.00000000
Br	-1.30074825	6.13286115	0.00000000

2,7-diBr-BTBTTO (anion)

-1 2

C	0.12255502	2.32629233	0.00000000
C	-1.03244857	1.51504177	0.00000000
C	-0.68366596	0.13652311	0.00000000
C	0.68366596	-0.13652311	0.00000000
C	-0.12255502	-2.32629233	0.00000000
C	1.03244857	-1.51504177	0.00000000
S	1.60210803	1.33820966	0.00000000
O	2.34655930	1.52913660	1.22369816
O	2.34655930	1.52913660	-1.22369816
S	-1.60210803	-1.33820966	0.00000000
O	-2.34655930	-1.52913660	-1.22369816
O	-2.34655930	-1.52913660	1.22369816
C	2.28128073	-2.14843836	0.00000000
H	3.18829292	-1.55766107	0.00000000
C	-0.07474886	-3.70201646	0.00000000
H	-0.97972522	-4.29541079	0.00000000
C	0.07474886	3.70201646	0.00000000
H	0.97972522	4.29541079	0.00000000
C	-2.34655930	3.52976434	0.00000000
H	-3.30709033	4.02681940	0.00000000
C	-2.28128073	2.14843836	0.00000000
H	-3.18829292	1.55766107	0.00000000
C	1.17979313	-4.29447691	0.00000000
C	-1.17979313	4.29447691	0.00000000
C	2.34655930	-3.52976434	0.00000000
H	3.30709033	-4.02681940	0.00000000
Br	1.31543367	-6.19215128	0.00000000
Br	-1.31543367	6.19215128	0.00000000
